# Vibrotactile feedback improves balance and mobility in patients with severe bilateral vestibular loss

**DOI:** 10.1007/s00415-018-9133-z

**Published:** 2018-12-05

**Authors:** Herman Kingma, Lilian Felipe, Marie-Cecile Gerards, Peter Gerits, Nils Guinand, Angelica Perez-Fornos, Vladimir Demkin, Raymond van de Berg

**Affiliations:** 1 Division of Balance Disorders, Department of Otorhinolaryngology and Head and Neck Surgery, Faculty of Health Medicine and Life Sciences, School for Mental Health and Neuroscience, Maastricht University Medical Centre, Maastricht, The Netherlands; 2 Faculty of Physics, Tomsk State University, Tomsk, Russian Federation; 3 Speech and Hearing Sciences Department, Lamar University, Rio de Janeiro, Brazil; 4 IDEE, Maastricht University, Maastricht, The Netherlands; 5 Service of Otorhinolaryngology and Head and Neck Surgery, Department of Clinical Neurosciences, Geneva University Hospitals, Geneva, Switzerland

**Keywords:** Vestibular prothesis, Balance, Sensory substitution, Bilateral vestibular loss

## Abstract

The impact of vibrotactile feedback of the gravity vector, provided by a “balance” belt worn around the waist, was evaluated in 39 patients with a severe bilateral vestibular loss, confirmed by extensive laboratory testing and suffering from a low quality of life, mainly due to imbalance. The mobility and balance score (MBS) of all patients prior to the use of the belt was equal or less than 5 out of a scale of 10. Thirty-one out of the 39 patients experienced the effect of the belt on their balance and mobility as positive in a preselection trial of 2 h in the hospital. The 31 positive responders then used the belt for 1 month in daily life. The average MBS increased significantly from 4.2 to 7.9 (paired *T* test, *T* = 9.82, *p* < 0.00001). Twenty-three out of 31 patients reported a benefit ranging from an improvement of 60–200% in their MBS. Eight patients did not experience any benefit. In summary, 23 out of 39 patients with a severe imbalance due to a bilateral vestibular loss experienced a clear benefit of vibrotactile feed = back in daily life. We conclude that vibrotactile feedback via the waist can serve as an effective prothesis for patients with severe bilateral vestibular loss to improve the quality of life.

**Electronic supplementary material:**

The online version of this article (10.1007/s00415-018-9133-z) contains supplementary material, which is available to authorized users.

## Introduction

Bilateral vestibular loss (BVL) is a chronic condition of which the causes can be ototoxic, infectious, traumatic, autoimmune or congenital. However, in approximately 30–50% of the cases no cause can be found [1]. The prevalence of severe BVL has been estimated at 81 in 100,000 persons, corresponding to 500,000 patients in Europe and the United States, and as many as 3 million worldwide [2, 3]. Due to a limited medical expertise and poor diagnostics worldwide, there is a substantial underestimation of the prevalence of BVL in the general population and a substantial delay before the diagnosis BVL is established [2]. BVL prevalence increases with age (presbyo-vestibulopathy), which becomes very apparent in developed countries with progressive aging of the populations. Patients with BVL complain mainly of imbalance, oscillopsia and a reduced dynamic visual acuity (DVA) [1, 4]. Various options to reduce symptoms have been studied [5–15]. There is robust evidence that vestibular rehabilitation can be effective in vestibular pathology and also in BVL [5, 10]. However, in severe BVL patients (including patients with bilateral areflexia), the impact of VR remains unfortunately limited in daily life balance and patients experience a sustained reduced quality of life [16]. Not many BVL patients improve in the long term [1–3]. In 2012 we implanted the first vestibular implant in humans [4, 16] to create a treatment option for the reduced DVA and imbalance in patients with severe BVL. But this approach is invasive, and still need substantial research to be clinically available. Already many years, research focussed on the development of non-invasive devices to restore balance per se, especially using feedback. Feedback by electrical, visual or auditory cues have been evaluated by several studies to improve balance in patients with severe vestibular deficits [17–25]. Electro-tactile feedback on the tongue has been evaluated extensively to restore imbalance [17, 22]. However, this is quite inconvenient for permanent chronic use. Auditory and visual feedback [18–20] for restorage of imbalance, interfere with the primary function of these senses. Continuous noisy galvanic stimulation at the level of the temporal bone (bilateral, retro-auricular) also provides a treatment option, which is suggested [25] to optimize residual vestibular resources in BVL by lowering the vestibular threshold to elicit balance-related reflexes. This intervention is only effective in the presence of a residual vestibular functionality, which applies for many patients with BVL. Vibrotactile feedback [21, 23, 24, 26, 27] can be used to increase somatosensory input to balance control. Vibrotactile feedback through the trunk is to this respect an intuitive approach and has been successfully used to reduce the need for visual navigation for special military forces, police officers and fire brigades, and to support spatial orientation for blind people [26, 28] and in industrial tele-manipulators [29]. Therefore, vibrotactile feedback to the body might be the best option to restore balance next to an implant. We, therefore, developed an ambulatory vibrotactile balance belt to increase and improve the proprioceptive perception of verticality in daily life, based on the same laboratory-based approach used by Wall III and colleagues [21, 26, 27]. The impact of continuous vibrotactile feedback in daily life is studied and evaluated in this study.

## Methods

### The vibrotactile belt

**Fig. 1:**
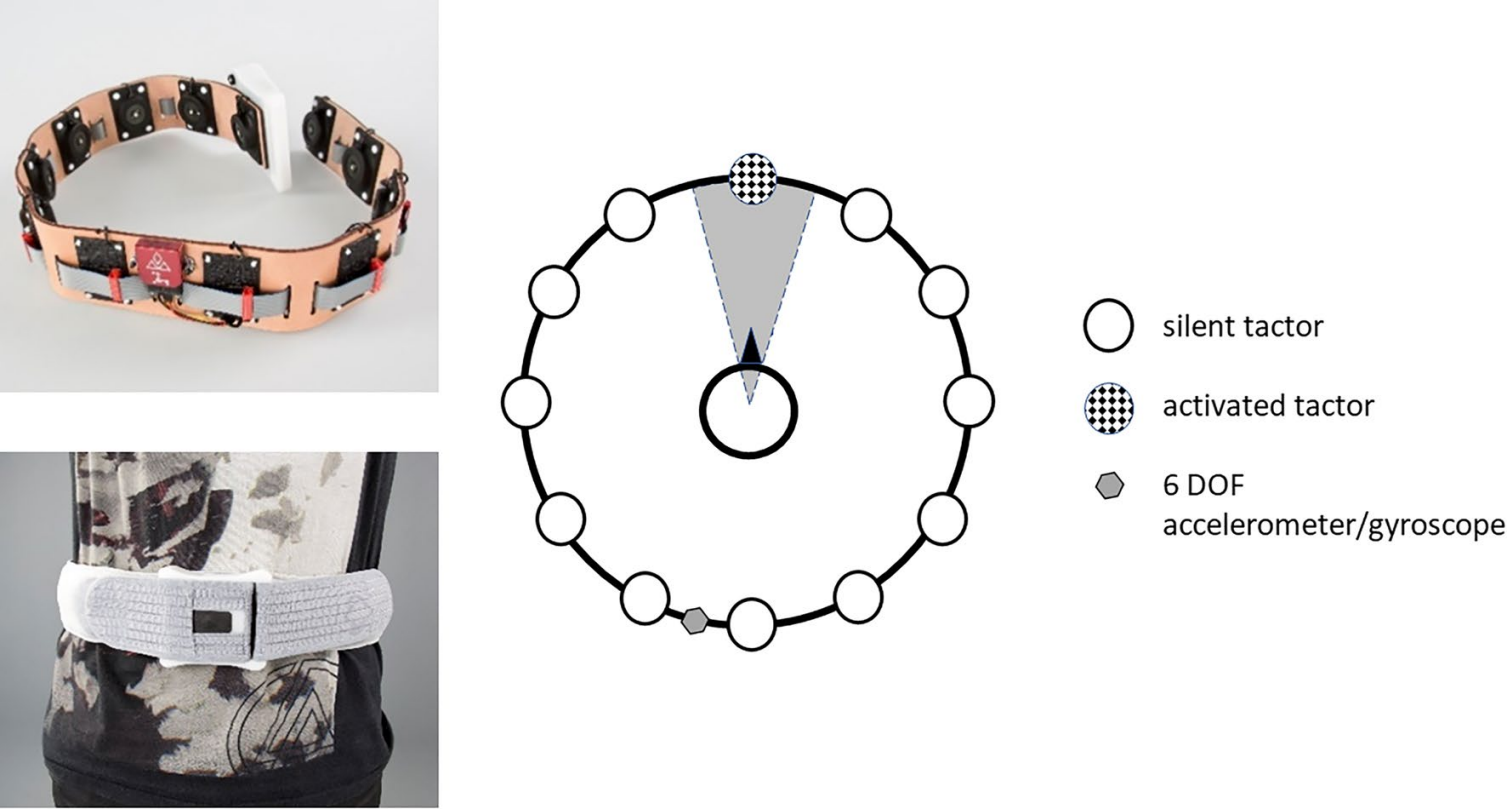
Balance belt. Upper left: top view without protecting sheet. Down left: balance belt worn around the waist in frontal view. Right: top view of belt activation pattern. The activation area is divided by sectors of 30 degrees, in each of them 1 tactor is placed. When the DOF-sensor (positioned at the back close to the spine) is inclined more than 2.5° degrees towards a specific sector, the associated tactor will be activated. When the sensor returns within a tilt angle of 1.5°, the tactor will become silent again. This 1° hysteresis is set to eliminate on-of oscillations

In contrast to other vibrotactile systems used to support rehabilitation programs, the current system is designed to be used permanently when standing or moving to improve balance. The current developed system is ambulant (battery life > 16 h of continuous use) using 12 tactors positioned in a belt worn around the waist (Fig. 1). The tactors are activated via a microprocessor based on the output of a 6DOF motion and tilt sensor also incorporated in the belt. The transfer function is fully programmable per tactor and accessible via Bluetooth.

### Pilot studies

The system was evaluated and further improved and developed based on the outcome of several pilot experiments since 2002. By this way the type of tactor, the material and construction of the belt were optimised to obtain an effective stimulation all over the waist, which appeared to be a big challenge. In a pilot study we observed that feedback over the waist proved to be superior to feedback to the head. In these pilot studies we also evaluated various outcome parameters in laboratory conditions (during and after use of the belt) to quantify the impact of vibrotactile feedback (sway angle, sway area, expert judgement of a double-blind gait analysis by video recording, Nintendo WII games, etc.). Improvement of all these parameters could be shown in these pilot studies, although with substantial variance. However, it was shown that the improvement was only significant if the patients wore the belt. Balance improvement disappeared almost immediately after the belt was removed or switched off. As a consequence and taking notice of a recent review using systems to aid and support rehab for patients with vestibular deficits [30], we decided to evaluate the clinical relevance of the use of the belt under daily life ambulatory conditions, and asked the patient to score their balance and mobility on a scale from 0 to 10 before and after 2 months of daily use of the belt. Although we are aware that this approach was subjective, we aimed to quantify the absolute effect of the belt in daily life.

### Patient selection

Patients were included after informed consent when they suffered from:

1. Severe Bilateral Vestibular Loss, defined as:
2. mean peak slow phase velocity of no more than 5°/s in bilateral bi-thermal caloric irrigations with water (30 and 44 degrees Celsius)
3. gain of less than 15% on rotatory chair test (sinusoidal stimulus, 0.1 Hz, peak velocity 90 °/s)
4. pathological head impulse test results for the horizontal and vertical canals.
5. Severe imbalance with a fear to fall and/or actual falls, despite undergoing various type of VR therapies previously
6. Quality of health score (QHS) as derived from the SF-36 < 40%.
7. Self-reported overall Mobility and Balance (MBS) score below 5 (test range 0–10).

The questionnaires SF-36 and MBS were submitted to patients during an interview at the Division of Balance Disorders at Maastricht University Hospital. The Short-Form Health Survey (SF-36) is a validated short-form health questionnaire composed of 36 items grouped into 8 variables: “physical functioning”, “role physical,” “bodily pain”, “general health”, “vitality”, “social functioning”, “role emotional” and “mental health,” assessing both mental and physical health [31, 32]. For each variable, item scores were coded, summed, and transformed on a scale from 0 (worst possible health state measured by the questionnaire) to 100% (best possible health state). It is a generic health measure, rather than disease-specific and used here as a general measure expressed in a quality of health score (QHS). The validated Dutch version of the SF-36 was used in this study [33].

The MBS was a number given by the patient on a scale from 0 to 10, as a subjective grading of his or her overall mobility and balance in daily life and is considered as a more specific outcome measure in this study. The intra-individual change of the MBS was used as a first approximation of the impact of the balance belt.

Patients with neurological, psychiatric or orthopaedic disorders, reduced proprioceptive sensitivity, or impaired vision were excluded.

Study 1: placebo controlled evaluation of vibrotactile feedback using 5 different feedback stimulation patterns.

Five different feedback patterns were applied to investigate the most effective feedback and to allow a placebo-controlled evaluation of the impact of the balance belt. The patterns were defined as follows:

1. As soon as the trunk inclination exceeded 2.5° relative to the gravity vector, the tactor towards which the trunk is inclined is activated: correct indication of the inclination direction. When the sensor returns within a tilt angle of 1.5°, the tactor will become silent again.
2. Placebo: as soon as the trunk inclination exceeds 2.5°, in 1 out of 3 times the “correct” tactor towards which the trunk is inclined is activated. However, in 2 out of 3 times one out of the other 11 tactors is activated (at random): indication of the correct inclination direction in only 33.3% of the cases. When the sensor returns within a tilt angle of 1.5°, any activated tactor will become silent again.
3. Placebo: as soon as the trunk inclination exceeds 2.5°, one out of the 12 tactors (at random) is activated: only feedback regarding the trunk inclination but no feedback of the inclination direction. When the sensor returns within a tilt angle of 1.5°, any activated tactor will become silent again.
4. Placebo: tactors are activated at random, with at random intervals of 1–3 s, without any correlation to the trunk inclination: at random vibration but no feedback regarding inclination.
5. Placebo: null mode: no vibration

We choose stimulus feedback pattern 1 for the tactor on the basis of the results of our pilot study: a 300 Hz sinusoidal signal delivered in sequences of 150 ms with a repetition rate of 4 Hz. Patients experience a vibratory pulse from a tactor when they tilt more than 2.5° towards that tactor. The stimulus is turned off when their tilt becomes less than 1.5° towards that sensor (Fig. 1).

A reset bottom allowed users to align the belt orientation with the gravity vector after mounting the device or when they needed to adjust their default body position (standing, sitting, car driving, etc.).

Based on the inclusion and exclusion criteria patients were selected and wore the belt for 1 day in the hospital using the standard feedback pattern 1. Five patients were selected that indicated that they experienced a clear improved balance and volunteered to participate in a double-blind “placebo-controlled” study using the belt programmed in 5 different feedback patterns, 1–5, defined as follows.

The patients wore the personalised belt with a specific feedback pattern for 6 weeks during all daily life activities. Each patient wore the same belt but with all 5 different feedback patterns for 6 weeks in random order. After each 6 weeks period, an interval of 2 weeks was taken without belt. The sequence of the 5 patterns was randomised over the 5 patients. The belts were programmed by a technician that annotated the codes of each belt. Patients, examiner and technician were blind regarding the specific feedback pattern used for a patient at any moment of the study. After 8 weeks the feedback pattern in the belt was changed according to the randomised protocol. At the start (*t* = 0), and after 2 (*t* = 2), after 4 (*t* = 4) and after 6 (*t* = 6) weeks the impact of the belt upon balance and gait was evaluated by the history, multiple tests and questionnaires.

The tests were:

- Romberg eyes open eyes closed (measuring sway area and sway velocity)
- Normal walking, with eyes open and eyes closed (video registration with blinded observation by 3 experts)
- Heel to toe walking, with eyes open and eyes closed (video registration with blinded observation by 3 experts)
- Ski-game on the Nintendo balance platform
- QOL score, Dutch version of the Short Falls Efficacy Scale-International (2007), Dizziness Handicap Inventory
- MBS score

Statistics was performed using SPSS, paired *t* tests.

#### Study 2

Based on these inclusion and exclusion criteria 39 patients were preselected. Subsequently, patients wore the belt for 2 h and were asked to indicate whether they experienced a clear benefit and wanted to participate in the 2-month study. Thirty-one patients, all indicating that they experienced a clear benefit, volunteered for participation in the study and signed an informed consent. Eight patients experienced no clear benefit of the belt during the 2 h try out and did not want to participate in the study.

After inclusion, all 31 patients were seen before and 1 month after the use of the belt in the outpatient clinic of the vestibular department by one of the primary investigators (Herman Kingma and Lilian Felipe). Before patients received the balance belt for a 1-month use, they had to indicate their mobility and balance score (MBS). They were asked to wear the belt continuously if they were standing or moving. A short instruction was given on how to use the belt. All patients started to use the belt without any problem and intuitively responded to the vibratory stimulus by adjusting their posture. After the trial, patients visited the outpatient department to return the belt. They were asked to indicate their MBS again. They were also actively asked to give additional comments about their subjective experiences with the belt.

### Statistics

Statistics was performed using SPSS, paired *t* tests.

### Ethical considerations

The procedures in this investigation were in accordance with the legislation and ethical standards on human experimentation in the Netherlands and in accordance with the Declaration of Helsinki (amended version 2013). Approval was obtained from the ethical committee of Maastricht University Medical Center. All procedures were performed at the Maastricht University Medical Center.

## Results

### Study 1

We were reluctant to share and describe the results of our first study. However, the clinical significance was clear. Analysis of the data, comparing the outcome parameters (including the MBS) of the 5 different feedback settings showed no significant differences (multiple *t* tests performed resulted in P values all below 0.03). The patients indicated that they did not experience any benefit of any setting and asked if they could retry the setting that was applied for the study inclusion. This was remarkable as all patients were included as responders using feedback pattern 1. The explanation for this result came after inspection of all belts. By a mistake in the belt programming, the intensity of the tactor vibration had been set 10 times lower than the setting at the inclusion. In fact, all 5 settings were placebo settings. Although patients felt vibrations. The conclusion was evident: at this intensity the belt had no significant impact even though patients and examiners were convinced that the belt should work at least in one of the specific settings. It was, therefore, decided to study the effect of feedback setting 1 without a placebo-controlled design and to focus on the MBS alone.

### Study 2

**Fig. 2:**
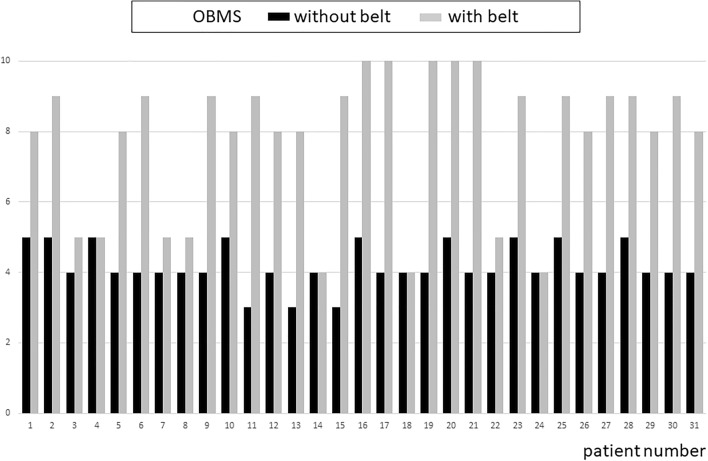
Individual pre- and post-MBS. Eight patients [3, 6, 7, 13, 16, 18, 20, 28] indicated no relevant improvement and did not apply for permanent use. The other 23 patients opted for permanent use

The average overall MBS increased significantly from 4.2 (pre-intervention) to 7.8 (post-trial) (paired *t* test, n-31, *T* = 9.82, *p* < 0.00001), see Table 1 and Fig. 2. Maximum relative MBS improvement was 200% of the pre-intervention MBS. Eight out of the 31 patients reported that they experienced no improvement and were disappointed by the belt, (see Fig. 2). They nevertheless wore the belt as agreed for 1 month. Twenty-three patients indicated a clear benefit during standing, walking and driving a car. Five patients started to use their bike again. The relative improvement in all patients ranged from 0 to 200%. When the 8 patients were excluded that reported no relevant benefit in the preselection stage, the variation among the positive patients was less: the improvement ranged from 60 to 200%.

**Table 1:** Mean, standard deviation and range of pre- and post-MBS in study 2

	Pre-MBS	Post-MBS	% improvement
Mean	4.2	7.8	89
SD	0.6	2.0	–
Range	3–5	4–10	0 to 200

### Subjective aspects: remarks of patients

All responders indicated that the positive effect of the belt disappeared immediately as soon as the belt was taken off. Patients experienced the battery time of 16 h as enough. Many patients indicated that their quality of life increased as they started to leave their homes again, started to go shopping again, visit friends and family again and had a reduced fear to fall. No specific improvement of DVA or oscillopsia was noticed by any patient. Some patients indicated a few drawbacks of the current belt design:

1. The belt was still too bulky
2. The activated tactors were audible, which irritated several patients or the people around them

## Discussion and conclusion

The treatment of patients with severe BVL is a real challenge for clinicians, especially given the wide spectrum of symptoms and complaints [34]. Several options are available like VR, technical devices to increase sensory substitution and vestibular implants. Especially VR is an interesting and relevant option to consider as it shows to be of crucial importance to facilitate central compensation and sensory substitution [10].

Vestibular rehabilitation.

In a recent review evaluating evidence for the impact of vestibular rehabilitation (VR) upon these complains, Kundacki et al. [5] found 4 studies of enough quality out of 376 electronic publication records. From these, Kundacki indicates that Hall et al. [6] observed an improvement of gaze stability by VR in patients with bilateral vestibular loss and that Yardley [7–9] found a reduction of complaints in patients with chronic dizziness as diagnosed by general practitioners. The impact of VR was also described in an excellent overview by Whitney et al. [10]. The various VR strategies and the principles of recovery (adaptation, habituation, etc.) are described. Whitney et al. indicates that VR is effective in BVL patients referring to the following studies:

1. Herdman et al. [11, 12] indicate that after VR patients had better dynamic visual acuity.
2. Krebs [13] found that after 8 weeks of VR patients walked 8% faster and, during paced gait and stair-climbing, walked with greater stability, evidenced by a 10% larger maximum moment arm and a 17% decreased double-support duration during gait and stair stance. However, the self-reported Dizziness Handicap Inventory scores did not differ significantly between control and active VR.
3. Gillespie and Minor [14]: bilateral vestibular loss was diagnosed in 35 patients. Improvement after vestibular rehabilitation therapy was noted in 18 patients (51%), whereas 12 (34%) showed little or no change and 5 (15%) were not available for follow-up. The patients without improvement were more likely to have a chronic disorder as a cause of the vestibulopathy and had more medical comorbidities, on average, when compared with those who improved. Lower gains and time constants on rotatory chair testing were also seen in the group that did not improve: so, VR is—in line with our clinical experience—less effective in case of severe BVL (low gains and time constants are strong indicators for severe loss).
4. Brown et al. [15]: On a population basis, statistically significant improvement was observed after physical therapy in several laboratory tests and clinically significant changes (reduction of complaints) were demonstrated by 33–55% of the patients. But no change was noted in the patients’ risk of falling, their number of falls, and the use of assistive devices. Vibrotactile feedback has been used successfully to optimise VR [30]. In conclusion, VR is clearly of value in vestibular pathology, but the impact of VR is unfortunately limited on daily life balance and mobility in patients with severe BVL or bilateral vestibular loss. Not many severe BVL patients improve in the long term enough by VR alone [1–3].
5. Vestibular implants.

A vestibular implant in humans [4, 16] is a promising treatment option to restore the DVA and balance. However, the positive impact of the VI as applied in 12 patients in our clinics (Geneva and Maastricht), was only examined when the patients were in the clinic [3, 4, 16, 35, 36]. Due to restrictions made by the medical ethics committee, the VI had to be switched off in daily life. Recently, three patients were implanted by the research group led by Della Santina in John’s Hopkins Hospital. In this study the patients can wear the activated implant in daily life. Promising preliminary results have been reported at various conferences. However, a VI is invasive, and still substantial research is needed to prove it’s benefit and to allow a broad clinical application and the 14 impact on the quality of life in BVL [37, 38].

Therefore, our research also focussed on the development of non-invasive devices to restore balance per se, especially using vibrotactile feedback as described in this study.

### Increasing vestibular sensitivity

As mentioned above, continuous noisy galvanic stimulation at the level of the temporal bone (bilateral, retro-auricular) provides a treatment option for BVL patients with residual vestibular functionality [25] by lowering the vestibular threshold. Further research is needed to develop a safe and comfortable ambulatory application and to investigate the impact in daily life on balance, oscillopsia, DVA and the quality of life in BVL [38].

### Vibrotactile feedback

Vibrotactile feedback [21, 23, 24, 26, 27] has been applied in laboratory conditions to increase somatosensory input to balance control. However, we learned that it was quite a challenge to develop an effective feedback system that could be worn in daily life [23, 24] and tested in daily life in this study.

This study evaluated the effect of the belt in a selected group of 2 h usage responders. It was observed that the belt had a substantial impact on the quality of life in patients. Our previous laboratory studies showed, in line with the literature, that vibrotactile feedback improves balance. The MBS was a first attempt to quantify the clinically relevant effect in the patient group picturing the daily life experience. The major improvement reported by the responders was on their leading symptom for inclusion: imbalance improved. No specific improvement of DVA or oscillopsia was reported. The pre-testing phase of 2 h in the hospital, to select the responders, seems to be an efficient procedure to identify patients that might benefit from the belt (23 out of 31 patients). All responders indicated that the positive effect of the belt disappeared immediately as soon as the belt was taken off. This in line with our observation during all our pilot and previous study that there is no learning effect in the severe BVL patients that lasts after the belt is detached. It must be noted that all patients had some type of VR to improve their balance and gait to a maximum before inclusion in this study. Eight patients did not experience any benefit in daily life, despite the fact that they were included on positive 2 first impression. This group of patients did not differ from the responders regarding (known) pathology, the pre-MBS score, applied VR, motivation, life style, gender, age, type of activities or profession. Further research is needed to find possible indicators for the therapy failure.

We considered to apply the extensive objective testing in study 2 as applied in study 1 (sway angle, sway area, expert judgement of a double-blind gait analysis by video recording, Nintendo WII games, etc.). However, based on the negative results of the 1st study we first wanted to show if any effect at all could be shown when wearing the belt for a longer time, placebo effect or not. On top of that we argued that it would make hardly any sense to objectify any effect in the laboratory if patients did not experience any improvement at all during or after 1-month use. So, we decided to first evaluate the subjective and clinical impact of the belt per se. Now, that we observed a clear clinical positive effect in the 2nd study, follow-up studies are scheduled to yield an objective picture of the actual treatment effects (sway angle, sway area, expert judgement of a double-blind gait analysis by video recording, Nintendo WII games, etc.), combined with a reproducibility and placebo controlled study of the subjective measure (MBS). In this follow-up study we will use an improved belt design based on the patient’s feedback in the 2nd study (less noisy and bulky).

We also evaluated the use of the belt in 11 patients with imbalance due to a variety of neurological disorders, including peripheral neuropathy or polyneuropathy. Unfortunately, none of these patients reported any benefit after using the belt for 2 h or more.

Special attention will, therefore, also be paid in the follow-up studies, to determine which patients (pathology, etc.) are best suited for the use of the belt, setting the optimal indication.

## Conclusion

We observed that a period of 2 h wearing the balance belt, allowed a good preselection of patients that might have a clear benefit of using continuous vibrotactile feedback. Twenty-three out of the 31 patients selected this way experienced a clear benefit in daily life, 8 no benefit at all (study 2). The lack of any effect of vibrotactile feedback at low intensities in different placebo settings, suggest that the effect quantified in the 2nd study is not placebo. The positive responders experienced a relatively big improvement in quality of life (60% or more on the MBS scale) and wanted to keep the belt and use it permanently.

## Electronic supplementary material

Below is the link to the electronic supplementary material.

Supplementary material 1 (XLSX 18 KB)
